# Canonical Wnt signaling affects calcium homeostasis in serum-treated AC16 cells through MLN-mediated SERCA2a regulation

**DOI:** 10.1093/jmcb/mjaf050

**Published:** 2025-12-05

**Authors:** Ang Li, Yuanyuan Shen, Zhenyan Li, Wenyu Jiang, Jie Su, Xiaomin Song, Lin Li

**Affiliations:** School of Life Science, Hangzhou Institute for Advanced Study, University of Chinese Academy of Science, Hangzhou 310024, China; School of Basic Medical Sciences, Dalian University of Technology, Dalian 116024, China; School of Life Science, Hangzhou Institute for Advanced Study, University of Chinese Academy of Science, Hangzhou 310024, China; School of Life Science, Hangzhou Institute for Advanced Study, University of Chinese Academy of Science, Hangzhou 310024, China; School of Life Science, Hangzhou Institute for Advanced Study, University of Chinese Academy of Science, Hangzhou 310024, China; School of Life Science, Hangzhou Institute for Advanced Study, University of Chinese Academy of Science, Hangzhou 310024, China; Key Laboratory of Multi-Cell Systems, Shanghai Institute of Biochemistry and Cell Biology, Center for Excellence in Molecular Cell Science, Chinese Academy of Sciences, University of Chinese Academy of Sciences, Shanghai 200031, China; School of Life Science, Hangzhou Institute for Advanced Study, University of Chinese Academy of Science, Hangzhou 310024, China; Key Laboratory of Multi-Cell Systems, Shanghai Institute of Biochemistry and Cell Biology, Center for Excellence in Molecular Cell Science, Chinese Academy of Sciences, University of Chinese Academy of Sciences, Shanghai 200031, China

**Keywords:** Wnt, cardiac calcium homeostasis, SERCA2a, MLN, AC16 cells

## Abstract

The canonical Wnt/β-catenin pathway critically regulates cardiac calcium homeostasis, yet its interplay with microenvironmental factors remains unclear. This study reveals that fetal bovine serum (FBS) treatment alters Wnt-mediated calcium dynamics in AC16 cardiomyocytes. While Wnt activation elevates cytosol calcium in serum-free conditions, FBS supplementation reverses this response: Wnt inhibitors (SFRP2, XAV939, and LF3) induce cytosol calcium accumulation, while the activators (LiCl and Wnt3a) lose efficacy. Mechanistically, FBS ablates RyR2 expression, uncoupling calcium-induced calcium release. Consequently, calcium handling shifts to SERCA2a-dependent regulation. We identify myoregulin (MLN) as a pivotal effector of Wnt/β-catenin signaling, with Wnt inhibition upregulating MLN to suppress SERCA2a activity. MLN knockdown (90% suppression) abolishes the effects of Wnt inhibitors on SERCA2a function and calcium distribution patterns. RyR2 reconstitution in FBS-treated cells restores calcium release but not Wnt activation responses, confirming the dominant role of MLN. Crucially, a combination of RyR2 overexpression and MLN depletion fully restores Wnt–calcium responses, phenocopying serum-free conditions. Our work establishes a serum-dependent regulatory axis where Wnt/β-catenin signaling maintains calcium homeostasis by repressing MLN, thereby preserving SERCA2a function. This FBS-induced shift mirrors pathological adaptations in heart failure, positioning MLN as a therapeutic target for calcium-handling disorders.

## Introduction

Cardiac contractility is fundamentally governed by precise cytosol calcium cycling, a process critically dependent on the coordinated function of key proteins within the sarcoplasmic reticulum (SR) membrane (Bers and Shannon, 2013; Bers, 2014; Terrar, 2022). Ryanodine receptor 2 (RyR2), the primary calcium release channel, mediates calcium-induced calcium release (CICR) essential for excitation–contraction coupling (Zima and Mazurek, 2016; Terrar, 2022). Conversely, sarco/endoplasmic reticulum calcium-ATPase (SERCA2a) actively pumps cytosol calcium back into the SR lumen, facilitating relaxation and replenishing SR calcium stores for subsequent contractions (Lyon et al., 2011; Liu et al., 2020; Bovo et al., 2023). Dysregulation of either RyR2 or SERCA2a function is a hallmark of cardiac pathologies, including hypertrophy and heart failure, underscoring the paramount importance of understanding the molecular mechanisms governing their activity and expression (Duan, 2010; Shareef et al., 2014; Sikkel et al., 2014; Dridi et al., 2023).

The canonical Wnt/β-catenin signaling pathway, a highly conserved developmental pathway, plays multifaceted roles in the adult heart, influencing processes ranging from hypertrophy and fibrosis to metabolism (Kaplan et al., 2019; Edwards et al., 2020; Han et al., 2022; Li et al., 2022). Emerging evidence, as well as our previous research, also implicates canonical Wnt signaling in the direct regulation of cardiac calcium homeostasis (Spinsanti et al., 2008; Wang et al., 2019; Li et al., 2025). Our previous work identified a specific mechanism whereby canonical Wnt signaling activation in cardiomyocytes promotes RyR2 activation through the upregulation of muscle-selective A kinase anchoring protein (AKAP6) (Li et al., 2025). AKAP6 functions as a scaffold protein facilitating PKA-mediated phosphorylation of RyR2 at S2808 (Ruehr et al., 2003; Kritzer et al., 2014), and we were the first to demonstrate *AKAP6* as a direct target gene of the canonical Wnt signaling (Li et al., 2025).

Fetal bovine serum (FBS), a ubiquitous component of cell culture media, profoundly influences cell morphology, proliferation, differentiation, and signaling pathways (Ferruzza et al., 2012; Fang et al., 2017; Dong et al., 2021; Wu et al., 2022). Notably, serum deprivation or supplementation can induce dramatic shifts in the gene expression profiles of cardiomyocytes and other cell types (Driesen et al., 2014; Khaleghi et al., 2014; Ranjan et al., 2021). While serum factors are known to modulate various signaling cascades, their specific impact on Wnt-mediated calcium regulation and the expression/function of core calcium-handling proteins such as RyR2 and SERCA2a remains elusive. Here, the AC16 cardiomyocyte cell line, maintained in standard FBS-free DMEM/F12 medium, exhibited calcium responses consistent with our previous findings. However, FBS supplementation reversed these responses. We identified the known SERCA2a inhibitor myoregulin (MLN) (Anderson et al., 2015) and RyR2 as the key mediators of this serum-dependent switch. FBS ablates RyR2 expression and establishes MLN as a critical Wnt/β-catenin-regulated inhibitor of SERCA2a, defining a novel regulatory axis essential for maintaining calcium homeostasis under serum-supplemented conditions. This discovery elucidates a new mechanism by which Wnt signaling governs cardiac calcium regulation and provides novel insights for potential therapeutic approaches targeting calcium-handling disorders.

## Results

### AC16 cells treated with FBS exhibit reversed responses to Wnt modulators

**Figure 1:**
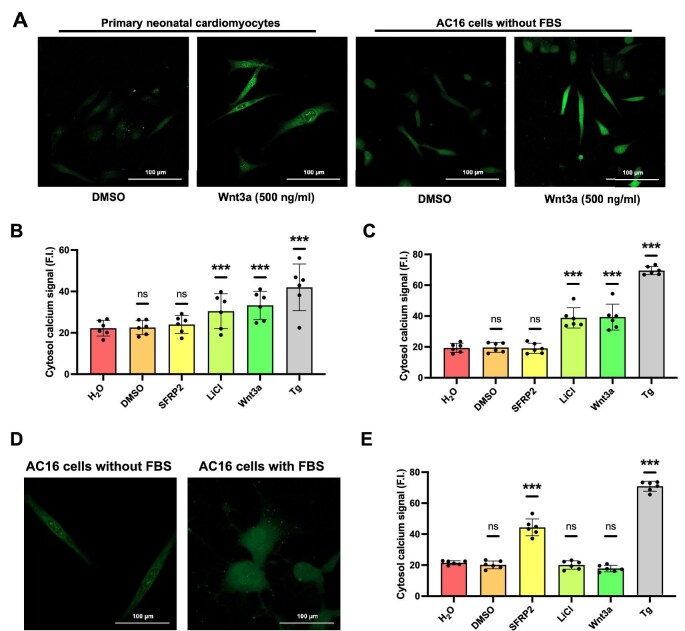
FBS treatment reverses the effects of Wnt modulators on cytosol calcium in AC16 cells. (**A**) Confocal images of primary neonatal cardiomyocytes and AC16 cells (without FBS) treated with or without Wnt3a for 6 h. Fluo4-AM dye was used to indicate cytosol calcium signals. Fluorescence intensity (F.I.) was calculated to represent the calcium level. (**B** and **C**) Cytosol calcium levels of primary neonatal cardiomyocytes and AC16 cells (without FBS) treated with the indicated compounds for 6 h. SFRP2: 10 μg/ml; Wnt3a: 500 ng/ml; LiCl: 20 mM. Tg (100 nM) was used as a positive control. (**D**) Confocal images of AC16 cells in the medium with or without 10% FBS for Fluo4-AM dye detection. AC16 cells without FBS exhibit cardiomyocyte-like fusiform morphology (left), while FBS supplementation induces morphological changes (right). (**E**) Cytosol calcium levels of AC16 cells in FBS medium treated with the indicated compounds for 6 h. Error bars indicate SD (*n* = 6).

Our previous study demonstrated that the canonical Wnt signaling activation disrupts calcium regulation in cardiac cells (Li et al., 2025). In this study, we initially tested the effects of Wnt activators and inhibitors on the cytosol calcium levels of primary neonatal cardiomyocytes from C57BL6 mice and the AC16 human cardiomyocyte cell line. The cells were preincubated with 2 μM of Fluo4-AM for 1 h, followed by 6 h incubation with different compounds, and then images were acquired at 488 nm excitation wavelength (Figure 1A). In standard (FBS-free) medium, the Wnt activator LiCl (20 mM) or Wnt3a (500 ng/ml) significantly upregulated cytosol calcium levels in both primary neonatal cardiomyocytes and AC16 cells, while the Wnt inhibitor SFRP2 (10 μg/ml) did not alter cytosol calcium levels (Figure 1B and C). Supplementing the DMEM/F12 medium with 10% FBS induced distinct morphological changes in AC16 cells. While cells in FBS-free medium displayed a characteristic fusiform shape typical of cardiomyocytes, those cultured in FBS-containing medium adopted a different morphology (Figure 1D). Of note, in FBS-containing medium, Wnt activators LiCl (20 mM) and Wnt3a (500 ng/ml) no longer altered cytosol calcium levels, while the Wnt inhibitor SFRP2 (10 μg/ml) increased cytosol calcium concentration (Figure 1E). The SERCA inhibitor thapsigargin (Tg, 100 nM) served as a positive control, elevating calcium as expected.

### Canonical Wnt signaling inhibition disrupts calcium homeostasis in FBS-treated AC16 cells

**Figure 2:**
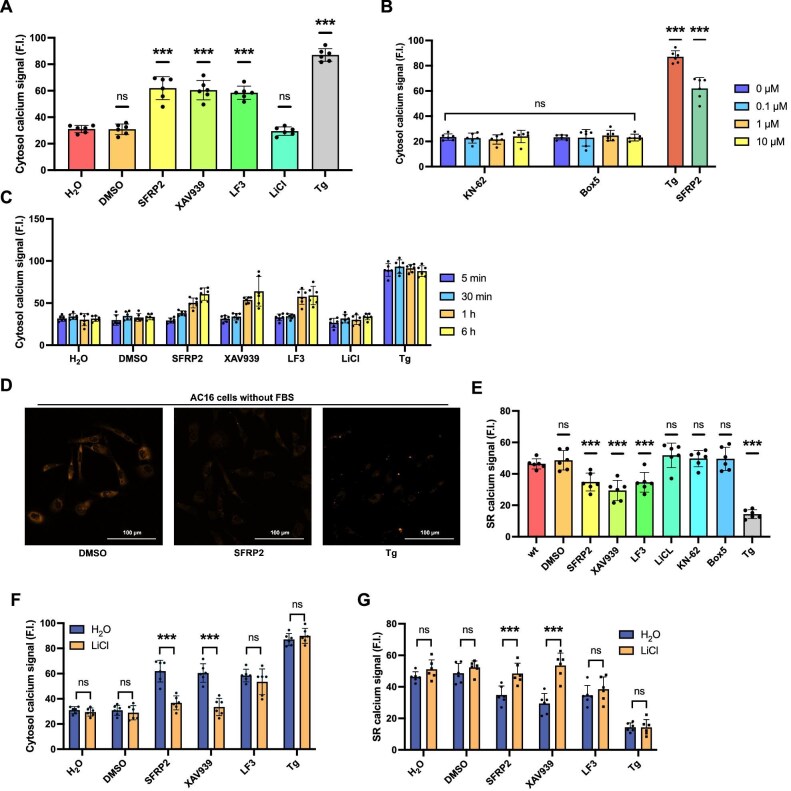
Canonical Wnt signaling inhibition disrupts calcium homeostasis in FBS-treated AC16 cells. (**A** and **B**) Cytosol calcium levels of AC16 cells in FBS medium treated with SFRP2 (10 μg/ml), XAV939 (10 μM), LF3 (1 μM), LiCl (20 mM), different amounts of KN-62 or Box5, and Tg (1 μM) for 6 h. (**C**) Cytosol calcium levels of AC16 cells in FBS medium treated with the indicated compounds for 5 min, 30 min, 1 h, and 6 h. (**D**) Confocal images of AC16 cells (without FBS) treated with DMSO, SFRP2, or Tg. R-CEPIA1er was used to indicate SR calcium signals. (**E**) SR calcium levels of AC16 cells treated with SFRP2 (10 μg/ml), XAV939 (10 μM), LF3 (1 μM), LiCl (20 mM), and Tg (1 μM) for 6 h. (**F** and **G**) Cytosol and SR calcium levels of AC16 cells treated with SFRP2 (10 μg/ml), XAV939 (10 μM), LF3 (1 μM), and Tg (1 μM) for 6 h, with LiCl (20 mM) added 15 min after each compound. Error bars indicate SD (*n* = 6).

To determine whether SFRP2-induced calcium dysregulation originated from the canonical (Wnt/β-catenin) or the non-canonical (Wnt/Ca²⁺) pathways, we treated FBS-exposed AC16 cells with two more mechanistically distinct canonical Wnt inhibitors: XAV939 (10 μM), which is a cytoplasmic tankyrase 1/2 inhibitor, and LF3 (1 μM), which is a nuclear β-catenin/TCF4 interaction blocker. Both inhibitors significantly increased cytosol calcium levels after 6 h, mirroring effects of SFRP2, while the canonical Wnt activator LiCl (20 mM) showed no effect (Figure 2A). We next assessed whether the non-canonical Wnt/Ca²⁺ pathway is involved by using Box5 (Wnt5a antagonist) and KN-62 (CaMKII phosphorylation inhibitor). Neither compound altered cytosol calcium concentrations at any tested dose (Figure 2B), indicating that SFRP2-mediated calcium accumulation operates independently of Wnt/Ca²⁺ signaling. Time-course analysis (5 min–6 h) revealed that canonical Wnt inhibitors (SFRP2, XAV939, and LF3) required ∼1 h to significantly elevate cytosol calcium levels, with early time points (≤30 min) showing no response and the time point of 6 h showing the maximal effects (Figure 2C). Since cytosol calcium overload typically reflects SR leakage, we monitored SR calcium using AC16 cells stably expressing the R-CEPIA1er biosensor. The cells were incubated with different compounds for 6 h, and then images were acquired at 561 nm excitation wavelength (Figure 2D, orange). Canonical Wnt inhibition (SFRP2/XAV939/LF3; 6 h) significantly decreased SR calcium levels (Figure 2E), confirming that the cytosol calcium accumulation results from SR calcium efflux. Results from the time-course analysis performed in AC16 cells stably expressing R-CEPIA1er were consistent with those obtained using the Fluo-4 AM dye (Supplementary Figure S1).

LiCl (20 mM) co-administration reversed SFRP2- and XAV939-induced calcium accumulation in both compartments but failed to rescue LF3-induced effects (Figure 2F and G), consistent with that LF3 and LiCl share nuclear targets (β-catenin/TCF4). Dose–response experiments to determine the competitive dosage effects between LiCl and LF3 were performed. LF3 (1 μM) did not show significant effect, while LiCl at higher concentrations adversely affected cell health, with concentrations exceeding 100 mM significantly inducing cell death (Supplementary Figure S2). Consequently, no effective competitive dosage could be identified. Together, these data demonstrate that the canonical Wnt/β-catenin signaling maintains calcium homeostasis in FBS-treated AC16 cells by preventing SR calcium leakage.

### FBS treatment ablates RyR2 expression and alters Wnt responsiveness in AC16 cells

**Figure 3:**
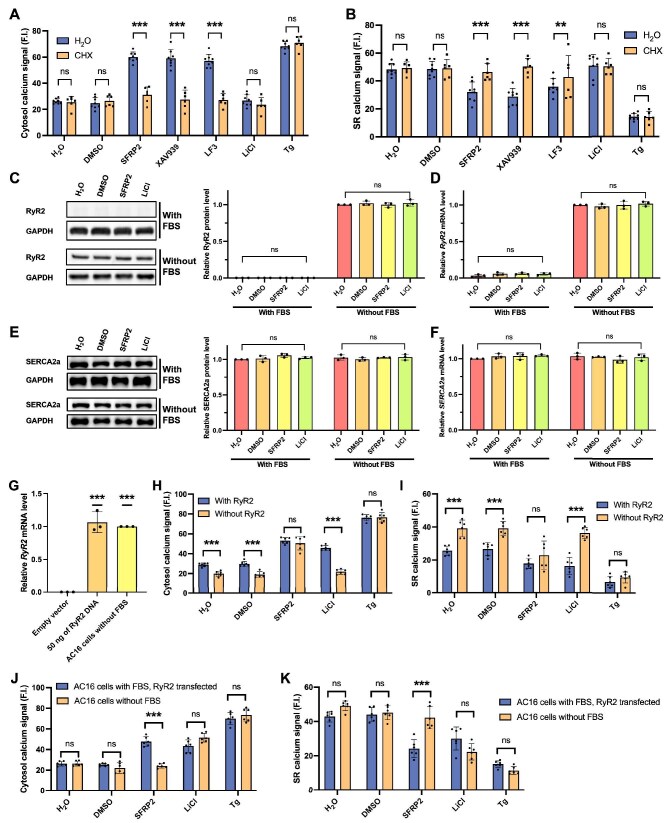
FBS-induced RyR2 ablation underlies impaired Wnt responsiveness in AC16 cells. (**A** and **B**) Cytosol and SR calcium levels of AC16 cells preincubated with H_2_O or CHX (1 μg/ml) for 15 min before treatment with Wnt modulators for 6 h. Error bars indicate SD (*n* = 6). (**C** and **E**) Western blot analysis of RyR2 and SERCA2a levels in AC16 cells in the medium with or without FBS treated with SFRP2 (10 μg/ml) or LiCl (20 mM) for 6 h. Error bars indicate SD (*n* = 3). (**D** and **F**) Relative *RyR2* and *SERCA2a* mRNA levels in AC16 cells with or without FBS treated with SFRP2 or LiCl for 6 h. Error bars indicate SD (*n* = 6). (**G**) Relative *RyR2* mRNA level for ‘RyR2-reconstituted’ cells compared to AC16 cells without FBS. (**H** and **I**) Cytosol and SR calcium levels of AC16 cells in FBS medium (labeled ‘Without RyR2’) and ‘RyR2-reconstituted’ cells in FBS medium (labeled ‘With RyR2’). (**J** and **K**) Cytosol and SR calcium levels of ‘RyR2-reconstituted’ cells in FBS medium and AC16 cells without FBS. Error bars indicate SD (*n* = 6).

Wnt inhibitors did not show any effects on cytosol calcium levels at early time points (≤30 min) (Figure 2C), implying that the canonical Wnt pathway might regulate cardiac calcium concentration through downstream effectors. To test this hypothesis, we used cycloheximide (CHX) to block protein synthesis in AC16 cells before treatment with Wnt modulators. The results showed that CHX reversed the effects of canonical Wnt inhibitors SFRP2, XAV939, and LF3 on the cytoplasm and SR calcium levels (Figure 3A and B), implying that canonical Wnt signaling affects cytosol calcium concentration by regulating downstream target expression.

**Figure 4:**
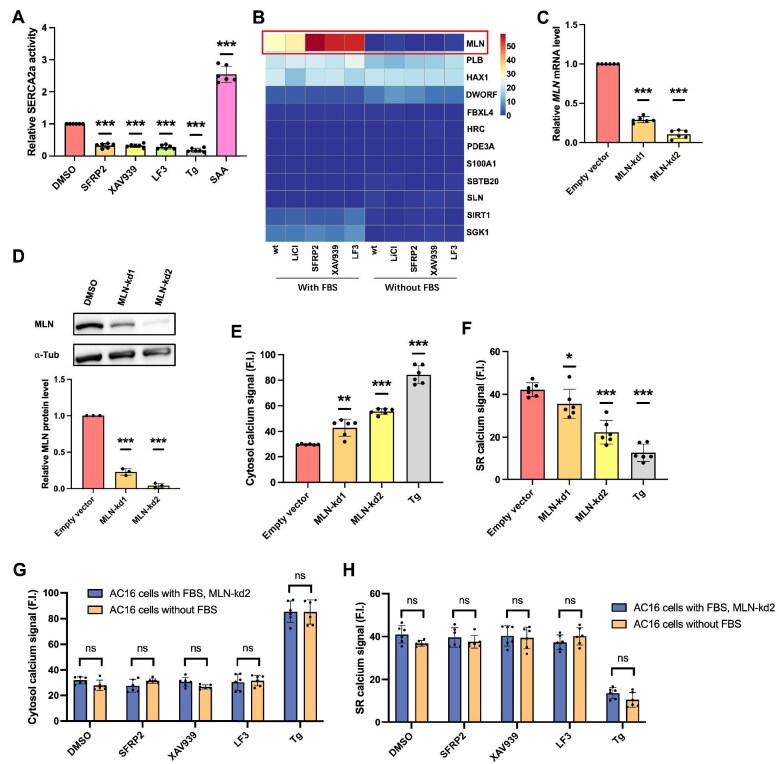
Wnt inhibition upregulates MLN expression to suppress SERCA2a activity. (**A**) Relative SERCA2a activity in AC16 cells in FBS medium. (**B**) Gene expression of known SERCA2a modulators in AC16 cells with or without FBS treated with different Wnt modulators (LiCl: 20 mM; SFRP2: 10 μg/ml; XAV939: 10 μM; LF3: 1 μM) for 6 h. (**C**) Relative *MLN* mRNA level in AC16 cells with FBS treatment. (**D**) Western blot analysis of MLN in AC16 cells with FBS treatment. (**E** and **F**) Cytosol and SR calcium levels of MLN-knockdown cell lines in FBS medium. (**G** and **H**) Cytosol and SR calcium levels of MLN-kd2 AC16 cells in FBS medium and AC16 cells without FBS. Error bars indicate SD (*n* = 6).

Given the central role of RyR2 and SERCA2a in cardiac calcium handling, we next examined their expression profiles. Strikingly, RyR2 was undetectable in FBS-treated AC16 cells regardless of Wnt modulator exposure (SFRP2 or LiCl), while RyR2 mRNA and protein expression remained robust and unaltered by Wnt modulators under FBS-free condition (Figure 3C and D), suggesting that the ablation of RyR2 protein by FBS treatment is likely mediated through post-transcriptional mechanisms such as enhanced degradation or suppressed translation. Furthermore, the basal upregulation of MLN in FBS-cultured cells (Figure 4B) may result from serum-induced partial suppression of canonical Wnt signaling that leads to derepression of MLN transcription. SERCA2a levels, however, were stable across all treatment groups (Figure 3E and F), indicating selective ablation of RyR2 specifically in FBS-exposed cells. To determine whether RyR2 loss underlies the aberrant Wnt responses, we stably expressed human RyR2 in FBS-treated AC16 cells (‘RyR2-reconstituted’), achieving expression levels comparable to FBS-free controls (Figure 3G). Reconstituted cells exhibited significantly higher basal cytosol calcium level (Figure 3H) and lower SR calcium level (Figure 3I) compared to those without RyR2 in FBS medium. Crucially, Wnt activation responses were restored: LiCl treatment significantly increased cytosol calcium level (Figure 3H) and decreased SR calcium level (Figure 3I), recapitulating the response pattern of FBS-free cells (Figure 3J and K). Notably, Wnt inhibition responses persisted in reconstituted cells, with SFRP2 further elevating cytosol calcium level and reducing SR calcium level (Figure 3J and K). Collectively, RyR2 ablation is necessary for the loss of Wnt activation responses in FBS-treated AC16 cells but insufficient to account for the acquired sensitivity to Wnt inhibition, indicating the involvement of additional FBS-induced regulatory mechanisms.

### Wnt inhibition upregulates MLN to suppress SERCA2a activity and mediate calcium dysregulation

Given the stable SERCA2a expression across various experimental conditions (Figure 3E and F), we hypothesized that the observed calcium dysregulation might result from altered SERCA2a activity. SERCA2a activity significantly declined following canonical Wnt inhibition with SFRP2, XAV939, or LF3 (Figure 4A). This reduced calcium-pumping activity directly corresponds to the observed cytosol calcium accumulation, as SERCA2a mediates calcium translocation from cytosol to SR lumen (Liu et al., 2020). Transcriptome analysis of FBS-treated AC16 cells exposed to Wnt modulators (LiCl: 20 mM; SFRP2: 10 μg/ml; XAV939: 10 μM; LF3: 1 μM; 6 h) identified *MLN* as a key differentially expressed gene, with basal *MLN* expression elevated in FBS-treated cells versus FBS-free controls and further upregulated by all canonical Wnt inhibitors (Figure 4B). Since MLN is a known direct inhibitor of SERCA2a (Anderson et al., 2015), these findings suggest a mechanistic cascade whereby Wnt inhibition induces MLN upregulation, thereby suppressing SERCA2a activity and driving cytosol calcium accumulation. The basal upregulation of MLN in FBS-cultured cells may result from serum-induced partial suppression of the canonical Wnt signaling, leading to derepression of MLN transcription.

To validate this mechanism, we generated MLN-knockdown cell lines in FBS-treated AC16 cells using shRNA, achieving ∼60% (MLN-kd1) and ∼90% (MLN-kd2) transcriptional suppression (Figure 4C) and marked protein reduction (in particular MLN-kd2) (Figure 4D). MLN depletion elevated basal cytosol calcium level (Figure 4E) and reduced SR calcium level (Figure 4F). Of note, MLN-kd2 abolished cellular calcium responses to Wnt inhibitors SFRP2, XAV939, and LF3 (Figure 4G and H), rendering FBS-treated cells phenotypically indistinguishable from FBS-free controls in their calcium responses. In addition, Wnt activators (Wnt3a and LiCl) did not alter cytosol or SR calcium levels in MLN-kd2 cells cultured with FBS (Supplementary Figure S3), which can be attributed to the loss of RyR2 expression in these cells. To assess whether SERCA2a inhibition or calcium dysregulation feeds back to modulate Wnt/β-catenin signaling, we examined the expression of canonical Wnt target genes (*c-Myc* and *CCND1*) in AC16 cells under various treatments. Neither SERCA2a inhibition (via thapsigargin) nor MLN overexpression altered the expression levels of these Wnt targets (Supplementary Figure S4), suggesting that the Wnt–MLN–SERCA2a axis operates unidirectionally under these conditions. These results indicate that MLN is necessary for transducing Wnt inhibition signals into SERCA2a-mediated calcium dysregulation in FBS-exposed AC16 cells.

### Combined MLN knockdown and RyR2 overexpression restores canonical calcium responses to Wnt modulators

**Figure 5:**
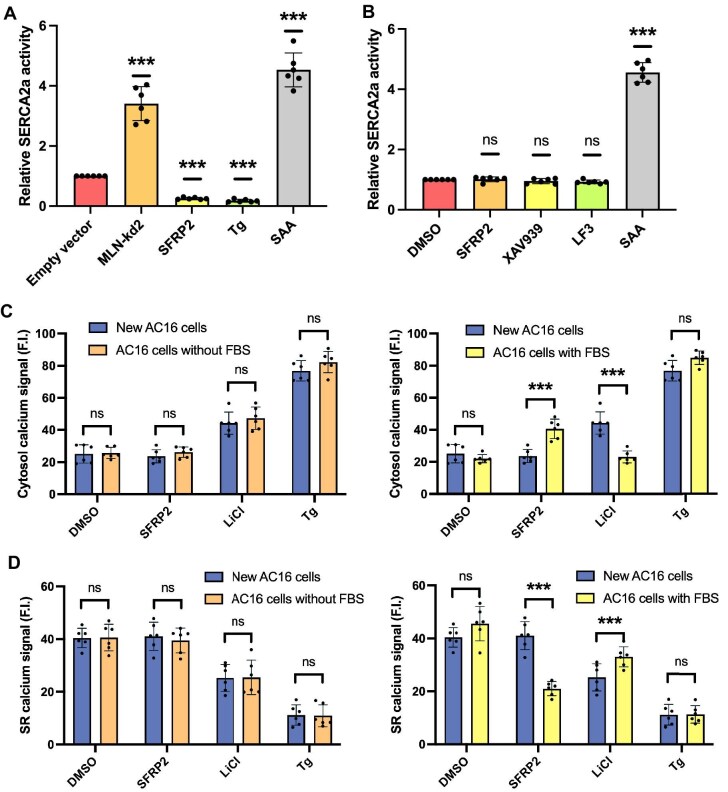
Combined MLN knockdown and RyR2 overexpression restores canonical Wnt-mediated calcium regulation in FBS-treated AC16 cells. (**A**) Relative SERCA2a activity in FBS-treated AC16 cells and MLN-kd2 cells under various treatments. Cells were treated with the SERCA2a activator SAA (1 μM), the Wnt inhibitor SFRP2 (10 μg/ml), or the SERCA2a inhibitor Tg (100 nM) for 6 h. MLN knockdown significantly increased basal SERCA2a activity and abolished SFRP2-induced suppression. (**B**) Relative SERCA2a activity in MLN-kd2 cells treated with canonical Wnt inhibitors (SFRP2: 10 μg/ml; XAV939: 10 μM; LF3: 1 μM) or the SERCA2a activator SAA (1 μM) for 6 h. (**C** and **D**) Cytosol and SR calcium levels in modified AC16 cells (‘New AC16 cells’) with combined MLN knockdown and RyR2 overexpression, compared to standard FBS-free AC16 cells and FBS-treated AC16 cells. Cells were treated with the Wnt activator LiCl (20 mM) or the Wnt inhibitor SFRP2 (10 μg/ml) for 6 h. Error bars indicate SD (*n* = 6).

To further elucidate the mechanistic role of MLN in Wnt-mediated calcium dysregulation, we assessed SERCA2a function following MLN knockdown in the MLN-kd2 cell line. SERCA2a activity was significantly elevated upon MLN knockdown (Figure 5A), consistent with MLN’s role as a direct inhibitor. The SERCA2a activator SAA (1 μM), which served as a positive control, further increased the activity of SERCA2a, while SFRP2 and the SERCA inhibitor thapsigargin suppressed activity as expected (Figure 5A). Crucially, in MLN-kd2 cells, canonical Wnt inhibitors (SFRP2, XAV939, and LF3) no longer suppressed SERCA2a activity, while SAA retained its stimulatory effect (Figure 5B). This confirms that MLN is essential for mediating Wnt inhibition-induced suppression of SERCA2a function.

We next investigated whether simultaneously ablating MLN (via knockdown) and restoring RyR2 expression (via overexpression) in FBS-treated AC16 cells could recapitulate the calcium response profile observed in AC16 cells cultured in the standard (FBS-free) condition. While these modified cells (‘New AC16 cells’) retained the FBS-induced morphological alterations (Supplementary Figure S5), their functional response to Wnt modulators was restored. As shown in Figure 5C and D, these cells exhibited calcium dynamics indistinguishable from FBS-free AC16 controls: Wnt activation (LiCl) increased cytosol calcium level and decreased SR calcium level, while Wnt inhibition (SFRP2) no longer disrupted calcium homeostasis. Wnt activation and inhibition were confirmed by the mRNA levels of known Wnt target genes (*c-Myc* and *CCND1*) (Supplementary Figure S6). Collectively, the combined intervention of MLN knockdown and RyR2 overexpression is sufficient to reverse the FBS-induced alteration in Wnt responsiveness and restore canonical calcium signaling patterns.

## Discussion

This study elucidates a novel regulatory mechanism by which canonical Wnt signaling maintains calcium homeostasis in AC16 cardiomyocytes under serum-supplemented conditions. FBS treatment alters cellular responses to Wnt modulators by ablating RyR2 expression and establishes MLN as a critical downstream effector of Wnt/β-catenin signaling. These results provide mechanistic insights into how microenvironmental factors influence cardiac calcium handling and identify MLN as a potential therapeutic target.

The paradoxical reversal of Wnt responses in FBS-treated cells represents a critical observation. Although canonical Wnt activation increased cytosol calcium levels under serum-free conditions, which is consistent with our previous reports (Li et al., 2025), this effect was abolished in FBS-containing medium. By contrast, Wnt inhibitors induced calcium accumulation only in FBS-treated cells. This serum-dependent switch correlates with the complete ablation of RyR2 expression, which is the primary calcium release channel in cardiomyocytes. The absence of RyR2 functionally uncouples the CICR machinery, shifting regulatory dominance to SERCA2a-mediated calcium uptake. This explains why SERCA2a regulation through MLN becomes the principal determinant of calcium homeostasis under these conditions. Our discovery showed that Wnt inhibition upregulates MLN expression, providing the first evidence linking the canonical Wnt signaling to this key SERCA2a regulator. MLN’s critical role was unequivocally demonstrated by the fact that its knockdown abolishes the effects of Wnt inhibitors on both SERCA2a activity and calcium distribution. The Wnt–MLN–SERCA2a axis functions independently of RyR2 status, as MLN regulation persists even after RyR2 reconstitution. Future studies are required to identify the specific β-catenin/TCF-regulated enhancers controlling the MLN transcription.

Elevated MLN expression and SERCA2a inhibition mirror changes observed in heart failure (Anderson et al., 2015; Appleby et al., 2024; Barry et al., 2024). Our findings here suggest that suppressed canonical Wnt signaling reported in failing hearts may contribute to disease progression through the MLN-mediated SERCA2a dysfunction. The serum-induced RyR2 loss in AC16 cells parallels the reduced RyR2 expression seen in pathological cardiac remodeling (Benitah et al., 2021), indicating that this model captures clinically relevant molecular adaptations. Targeting the Wnt–MLN axis could therefore offer novel therapeutic strategies for calcium-handling pathologies. Interestingly, a previous study reported that ZFAS1, another endogenous inhibitor of SERCA2a, positively regulates the Wnt/β-catenin pathway (Zou et al., 2021). While ZFAS1 appears to enhance Wnt signaling, our data indicate that MLN is a downstream target of Wnt repression and does not feedback to modulate Wnt activity. This suggests that different SERCA2a inhibitors may be embedded within distinct regulatory circuits. Further studies are needed to explore potential crosstalk between ZFAS1 and MLN, as well as whether they function in parallel or convergent pathways. While this study establishes MLN as a key Wnt signaling effector, the precise mechanism of FBS-induced RyR2 ablation remains to be elucidated. In addition, *in vivo* validation in cardiac-specific β-catenin or MLN knockout models is needed, and the physiological relevance of serum concentrations used in this study requires proper contextualization.

From a therapeutic perspective, our work positions the Wnt–MLN–SERCA2a axis as a potential target for intervention. Strategies aimed at inhibiting MLN or disrupting its interaction with SERCA2a could reverse the SERCA2a dysfunction that is a hallmark of heart failure, independent of RyR2 status. Furthermore, modulating Wnt signaling to suppress MLN expression presents an alternative avenue for restoring calcium homeostasis. Therefore, this study not only elucidates a serum-dependent regulatory switch but also identifies a clinically relevant pathway whose manipulation may offer new strategies for treating calcium-handling disorders in heart disease.

## Materials and methods

### Cloning of vectors

R-CEPIA1er sequence was cut and pasted with *Nhe*I and *Not*I into the pcDNA3.1 vector with ampicillin resistance. shMLN was amplified by PCR with the following primers and then cloned into the pLKO.5 vectors: shMLN-1: 5′-CCGGTGATAATTCTGGATATGACTCTCGAGAGTCATATCCAGAATTATCTTTTTG-3′ (forward) and 5′-AATTCAAAAAGATAATTCTGGATATGACTCTCGAGAGTCATATCCAGAATTATCA-3′ (reverse); shMLN-2: 5′-CCGGTACTGAGTCATGAAACTTCTAGACTCGAGTCTAGAAGTTTCATGACTCAGTTTTTTG-3′ (forward) and 5′-AATTCAAAAAACTGAGTCATGAAACTTCTAGACTCGAGTCTAGAAGTTTCATGACTCAGTA-3′ (reverse). shRNA constructs were transiently transfected into AC16 cells, selected under 5 μg/ml of puromycin for 2 weeks, and then harvested for further experiments. Human RyR2 expressed in pcDNA3.1 was a kind gift from Zhang Ping's lab.

### Cell culture and transfections

AC16 cells were purchased from Meisen Cell and cultured in DMEM/F12 (Sigma) with or without 10% FBS. AC16 cells were transfected using Lipofectamine 3000 (Thermo Fisher Scientific). R-CEPIA1er constructs were expressed using the mammalian expression vector pcDNA3.1 with G418 resistance. AC16 cells were plated at 2 × 10^6^ cells/dish in 10-cm dishes 24 h before transfection. To select a stable cell line, 2 days after transfection, 1500 μg/ml of G418 antibiotic was added to the growth medium. Seven days after the antibiotic selection, the remaining cells were enriched and passaged. After 3 weeks of culture, there were 1 × 10^8^ cells, generating a stable clone expressing the biosensor at high levels. The stable cell line was maintained using DMEM/F12 medium and 1000 μg/ml G418.

### Fluo4-AM-mediated cytosol calcium detection

AC16 cells were plated in 24-well plates at 1 × 10^5^ cells/well. For experiment, 2 μM of Fluo4-AM (Beyotime, S1060) in Krebs buffer (140 mM NaCl, 4 mM KCl, 1 mM MgCl_2_, 1 mM CaCl_2_, 10 mM glucose, and 5 mM HEPES, pH 7.4) was added to each well. After 1 h incubation of Fluo4-AM, each well was washed three times with 500 μl of Krebs buffer. Then, various compounds, including SFRP1/2 (MedChemExpress, HY-P74554 and HY-P77835), XAV939 (MedChemExpress, HY-15147), LF3 (MedChemExpress, HY-101486), LiCl (Sigma, 656984), thapsigargin (MedChem-Express, HY-13433), KN-62 (MedChemExpress, HY-13290), Box5 (MedChemExpress, HY-123071), and CHX (MedChem-Express, HY-12320), at different concentrations, were added to the cells. Images were acquired at a 488-nm excitation wavelength under a Zeiss confocal microscope (ZEISS LSM 900). The fluorescence intensity was calculated and represented the cytosol calcium level.

### R-CEPIA1er-mediated SR calcium detection

AC16 cells stably expressing R-CEPIA1er were seeded in 24-well plates at 1 × 10^5^ cells/well. The medium was replaced with Krebs buffer, and different compounds were added and incubated for 6 h. Images were acquired at a 561-nm excitation wavelength under a Zeiss confocal microscope. The fluorescence intensity was calculated and represented the SR calcium level.

### Western blotting

Cell lysates were separated using 4%–12% SDS–PAGE (Invitrogen) and transferred onto nitrocellulose membranes (Millipore). Membranes were blocked with 5% non-fat dry milk and incubated with primary antibody overnight at 4°C. Monoclonal anti-SERCA2a antibody (ABclonal, A11692), monoclonal anti-RyR2 (Abcam, ab224803), polyclonal anti-MLN (Thermofisher, MYREGLN-101AP), monoclonal anti-tubulin antibody (Abcam, ab7291), and monoclonal anti-GAPDH (Abcam, ab8245) were used. Blots were visualized using HRP-conjugated secondary antibodies.

### Real-time PCR

Total RNA was extracted using a TRIzol kit (Invitrogen, Cat#15596026). Reverse transcription was performed using the SuperScript III First-Strand Synthesis System (Thermo Fisher Scientific, Cat#18080051) according to the manufacturer’s instructions. Quantitative real-time PCR was performed using SYBR Premix Ex Taq (Toyobo, Cat#QPK-201) on a QuantStudioTM6 Flex Real-Time PCR System (Applied Biosystems). Real-time PCR data were analyzed by the comparative CT method using β-actin as the normalization control. Primer sequences for *RyR2*: 5′-ACAACAGAAGCTATGCTTGGC-3′ (forward) and 5′-GAGGAGTGTTCGATGACCACC-3′ (reverse). Primer sequences for *SERCA2a*: 5′-CATCAAGCACACTGATCCCGT-3′ (forward) and 5′-CCACTCCCATAGCTTTCCCAG-3′ (reverse). Primer sequences for *MLN*: 5′-GAGACCACCTGTGCTGAATA-3′ (forward) and 5′-TATTCAGCACAGGTGGTCTC-3′ (reverse). Primer sequences for *c-Myc*: 5′-GCTGTTTGAAGGCTGGATTTC-3′ (forward) and 5′-GATGAAATAGGGCTGTACGGAG-3′ (reverse). Primer sequences for *CCND1*: 5′-GCTGGCCATGAACTACCTGGA-3′ (forward) and 5′-TCCATTTGCAGCAGCTCCTC-3′ (reverse).

### RNA sequencing

RNA sequencing (RNA-seq) was performed for AC16 cells treated with canonical Wnt inhibitors. AC16 cells was seeded in 6-well plates and cultured for 1 day. After 3 h incubation with SFRP2 (10 μg/ml), XAV939 (10 μM), LF3 (10 μM), and LiCl (20 mM), cells were harvested with TRIzol (Sigma). RNA-seq and data analysis were carried out by LC-Bio Technology Co. Ltd.

### SERCA2a activity assay

AC16 cells were centrifuged at 300× *g*, washed three times in phosphate-buffered saline (PBS, with no magnesium or calcium added; Thermo Scientific), and resuspended in homogenization buffer (0.5 mM MgCl_2_, 10 mM Tris–HCl, pH 7.5, DNase I, and protease inhibitor) at 1 × 10^7^ cells/ml. The cells were then incubated on ice for 10 min and lysed with a Tissumizer (Tekmar SDT-1810) with three 30-sec bursts. After each burst, a 5-min incubation on ice was performed. Homogenization was confirmed by microscopy. After homogenization, 2× sucrose buffer (1 mM MOPS, 500 mM sucrose, and protease inhibitor) was added to obtain a final cell concentration of 2 mg total protein/ml. An NADH-linked ATPase assay Kit (MEIAOBIO, MO-P37303R) was used to measure SERCA2a activity in 96-well microplates on a microplate reader.

### Statistical analysis

Data are presented as mean ± standard deviation (SD). Statistical values from Fluo4-AM and R-CEPIA1er assays were calculated from a minimum of eight separate experiments (*n* = 8). Statistical values from SERCA2a activity assays were calculated from a minimum of three separate experiments (*n* = 3). Two-group comparisons were performed by Student’s *t*-test (*P < 0.05, **P < 0.01, ***P < 0.001).

## Supplementary Material

mjaf050_Supplemental_File
