# Supplementary material for: Canonical Wnt signaling affects calcium homeostasis in serum-treated AC16 cells through MLN-mediated SERCA2a regulation
Source: J Mol Cell Biol. 2025 Dec 5;17(10):mjaf050. doi: 10.1093/jmcb/mjaf050 (PMC13093114; doi:10.1093/jmcb/mjaf050)
Supplement: mjaf050_Supplemental_File [file mjaf050_supplemental_file.pdf]

SUPPLEMENT INFORMATION

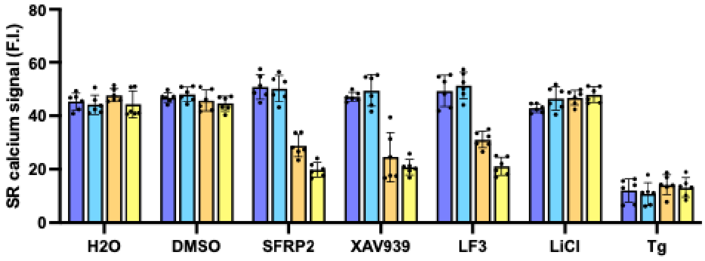

Supplementary Figure S1. Time-correlated Wnt/β-catenin inhibitors effects. Calcium signal images were acquired at 5 min, 30 min, 1 h and 6 h time points. Fluorescence intensity was calculated and represents for the SR calcium level.

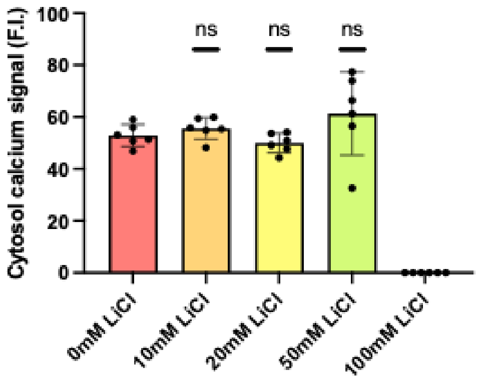

Supplementary Figure S2. Cytosolic and SR calcium signal with LiCl rescue determined by Fluo4-AM. AC16 cells were plated in 24-well plates at $1*10^5$ cells/well. One hour before the experiment, 2 µM of Fluo4-AM in Krebs buffer was added to each well. LF3 (1 µM) were added for 6 h, and different amount of LiCl was added to the system 15 min after each compound. Error bars indicate SD (n = 6).

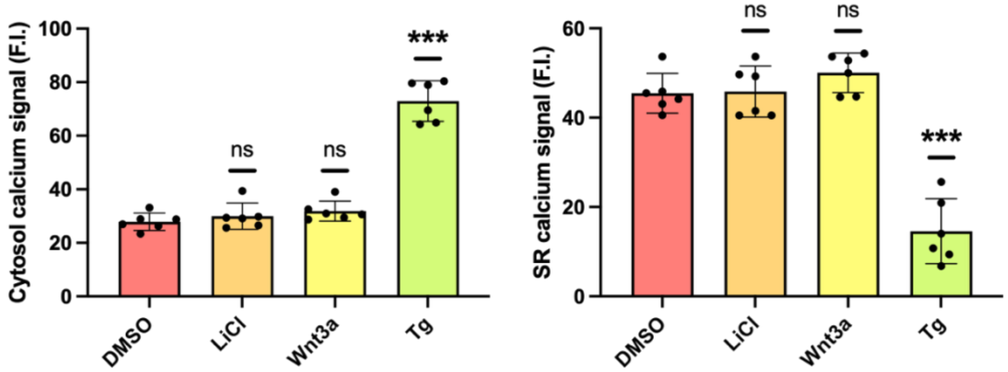

Supplementary Figure S3. Cytosol and SR calcium levels of MLN-kd2 AC16 cells with FBS-contained medium. Wnt activators (LiCl: 20 mM, Wnt3a: 500 ng/mL) was incubated for 6 hours. Tg (100 nM) was used as control. Error bars indicate SD (n = 6).

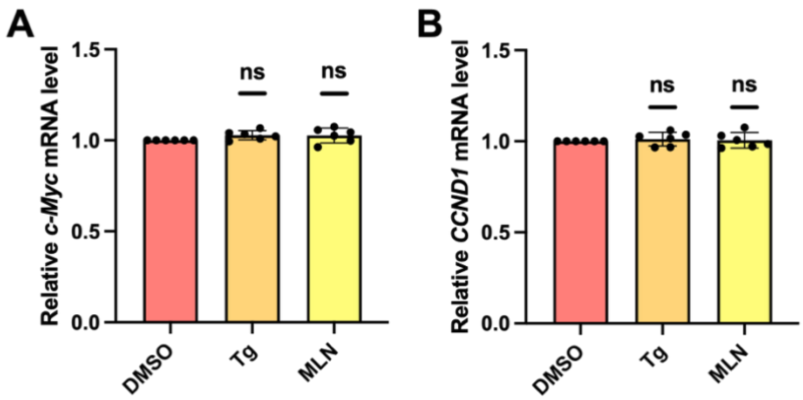

Supplementary Figure S4. Tg or MLN did not alter the expression of Wnt/β-catenin target genes in AC16 cells without FBS. **(A** and **B)** Relative mRNA levels of *c-Myc* and *CCND1*. Error bars indicate SD (*n* = 6).

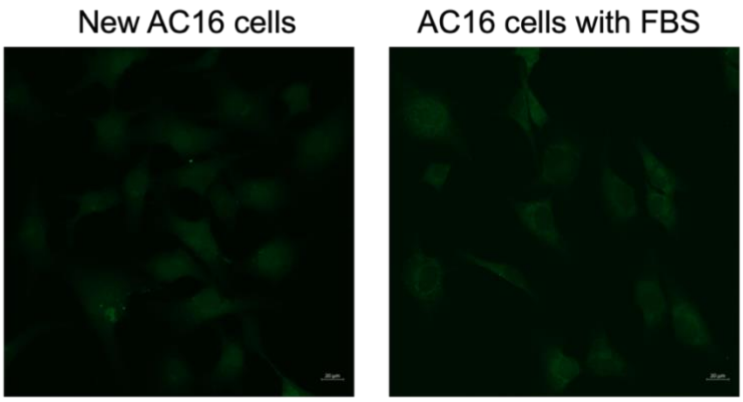

Supplementary Figure S5. Morphological persistence in genetically modified AC16 cells under FBS supplementation. Confocal microscopy images comparing wild-type AC16 cells (left, DMSO group) and genetically modified "New AC16 cells" (right, DMSO group; with combined MLN knockdown and RyR2 overexpression) cultured in FBS-containing medium.

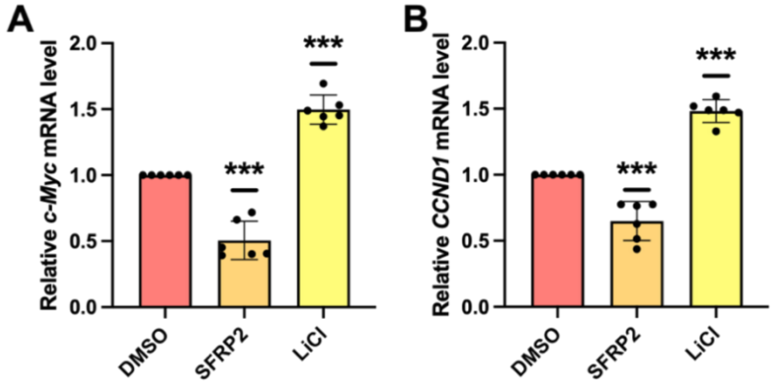

Supplementary Figure S6. Canonical Wnt modulators regulate the expression of Wnt/β-catenin target genes in AC16 cells. (A and B) Relative mRNA levels of *c-Myc* and *CCND1*. Error bars indicate SD (n = 6).
